# Task sharing in Zambia: HIV service scale-up compounds the human resource crisis

**DOI:** 10.1186/1472-6963-10-272

**Published:** 2010-09-17

**Authors:** Aisling Walsh, Phillimon Ndubani, Joseph Simbaya, Patrick Dicker, Ruairí Brugha

**Affiliations:** 1 Department of Epidemiology and Public Health Medicine, Division of Population Health Sciences, Royal College of Surgeons in Ireland, Dublin, Ireland; 2 Institute of Economic and Social Research, University of Zambia, Lusaka, Zambia; 3 Department of Global Health Development, Faculty of Public Health and Policy, London School of Hygiene and Tropical Medicine, London, UK

## Abstract

**Background:**

Considerable attention has been given by policy makers and researchers to the human resources for health crisis in Africa. However, little attention has been paid to quantifying health facility-level trends in health worker numbers, distribution and workload, despite growing demands on health workers due to the availability of new funds for HIV/AIDS control scale-up. This study analyses and reports trends in HIV and non-HIV ambulatory service workloads on clinical staff in urban and rural district level facilities.

**Methods:**

Structured surveys of health facility managers, and health services covering 2005-07 were conducted in three districts of Zambia in 2008 (two urban and one rural), to fill this evidence gap. Intra-facility analyses were conducted, comparing trends in HIV and non-HIV service utilisation with staff trends.

**Results:**

Clinical staff (doctors, nurses and nurse-midwives, and clinical officers) numbers and staff population densities fell slightly, with lower ratios of staff to population in the rural district. The ratios of antenatal care and family planning registrants to nurses/nurse-midwives were highest at baseline and increased further at the rural facilities over the three years, while daily outpatient department (OPD) workload in urban facilities fell below that in rural facilities. HIV workload, as measured by numbers of clients receiving antiretroviral treatment (ART) and prevention of mother to child transmission (PMTCT) per facility staff member, was highest in the capital city, but increased rapidly in all three districts. The analysis suggests evidence of task sharing, in that staff designated by managers as ART and PMTCT workers made up a higher proportion of frontline service providers by 2007.

**Conclusions:**

This analysis of workforce patterns across 30 facilities in three districts of Zambia illustrates that the remarkable achievements in scaling-up HIV/AIDS service delivery has been on the back of sustained non-HIV workload levels, increasing HIV workload and stagnant health worker numbers. The findings are based on an analysis of routine data that are available to district and national managers. Mixed methods research is needed, combining quantitative analyses of routine health information with follow-up qualitative interviews, to explore and explain workload changes, and to identify and measure where problems are most acute, so that decision makers can respond appropriately. This study provides quantitative evidence of a human resource crisis in health facilities in Zambia, which may be more acute in rural areas.

## Background

Zambia is one of 57 countries worldwide experiencing a health worker shortage crisis, which threatens its response to HIV/AIDS and other priorities [1]. In 2006, more than 50% of rural health centres had only one qualified staff member, numerous facilities had no staff, and thirteen districts did not have a single doctor [2]. Health workforce responses, such as task shifting, have been proposed to combat the crisis [3,4]. The Government of Zambia established a Human Resources Task Force in 2004, which produced the Human Resources for Health Strategic Plan 2006-2010 to address staff shortages [2]. The Plan aims "to provide a framework to guide and direct interventions, investments and decision making in the planning, management and development of human resources for health" [2]. Twenty years into the HIV pandemic, 13.1% of Zambian adults aged 15-49 are HIV positive [5], with a higher prevalence in urban (20%) than rural (10%) areas, where almost two thirds (64%) of the population lives [6]. Despite health worker shortages, Zambia has achieved a remarkable scale-up in the delivery of HIV/AIDS services, 2005-07 (see Table 1). In 2008, the numbers on antiretroviral treatment (ART) increased by almost a further 50% to 225,634 [7].

**Table 1:** Selected HIV/AIDS indicators in Zambia, 2005 - 2007

Indicator	2005	2006	2007
Population (in millions)	11.4	11.8	12.2

Adult HIV prevalence% (aged 15-49)	13.9	13.5	13.1

HIV prevalence in pregnant women (%)	19.1	19.1	19.3

Number (%) of adults and children with advanced HIV infection receiving ART	39 351	80 030 (32.9)	149 199 (50.5)

Number (%) of pregnant women needing and receiving ART to reduce the risk of mother to child transmission (PMTCT)	No data	25 578 (29.7)	35 314 (39.1)

Number(%) of women and men 15-49 who received a test in the last 12 months and knew their results	(15.6)	234 430 (15.4)	254 585 (15.4)

Number of sites providing ART	107	156	322

Number of sites providing PMTCT	67	307	678

Number of sites providing HIV Counselling and Testing (VCT)	No data	883	1028

% of adults and children with HIV still alive 12 months after initiation of ART	No data	89.6	87.6

Source: Zambia Country UNGASS Report 2008 (Ministry of Health Zambia, National HIV/AIDS Council of Zambia, 2008)

Zambia has benefited from high levels of external funding for HIV/AIDS control, which increased from US$6 in 2003 to US$10 per capita in 2006 [8]. The proportion of funding to HIV/AIDS from external sources rose from 70% to 74% during this period. The largest external funder was the US President's Emergency Plan for AIDS Relief (PEPFAR) contributing 50%, followed by the Global Fund to Fight AIDS, TB and Malaria, at 16% [8]. These two initiatives, which are termed Global Health or HIV/AIDS Initiatives (GHIs) [9], have used different strategies to support HIV/AIDS control. The Global Fund has used a country-led process, where country coordination mechanisms with broad stakeholder membership decide priorities and prepare and submit proposals to the Fund; whereas PEPFAR has used a top-down planning approach where it has dictated priorities [8,10]. Both provided funding for in-service training of health workers - staff training for Voluntary Counselling and Testing (VCT) and PMTCT were components of Zambia's successful Round 1 and Round 4 Global Fund grants. However, neither has contributed to increasing the pool of health workers.

The authors conducted a study of the effects of GHIs on the Zambian health system, in 2007-08, focusing on the effects of GHIs at district and facility levels, where there had been little published empirical research between 2002 and 2007 [11]. In this paper we define a GHI as: "a blueprint for financing, resourcing, coordinating and/or implementing disease control across at least several countries in more than one region of the world" [9]. Previous studies on the effects of GHIs on human resources for health in Zambia have focused on national level effects [10,12] and used qualitative methods to study national and sub-national effects [13].

There are several ways of measuring work and defining workload [14-16]. The definition used by Cirrin et al (2003) - 'activities required and performed related to the provision of services' - fits best with the findings we present [16]. Five generic methods are utilised in the workload measurement literature: personnel-to-population ratio method, health needs method, utilisation based method, service demand and service target method [14]. In practice, workforce planners use a combination of approaches [17,18], although the service target method is currently most favoured by the WHO [18]. This paper uses two workload measures: firstly a personnel-to-population ratio method, and secondly a ratio based utilisation method (ratio of staff numbers to activity measures), which has been used by others [14]. Workload depends on a variety of factors, such as organisation of service delivery, competency of staff, staff motivation, availability of equipment, infrastructure and drugs. While sub-national studies in Africa have shown the importance of assessing the impact of HIV services on workload [19,20] those assessing district level workload in Zambia are scarce, and where they have been carried out they do not use commonly used workload measurement methods [21,22].

The benefits of task shifting have been promoted in recent years, both globally [3,23] and in Zambia [24,25], as a strategy to compensate for health worker shortages. In task shifting, "specific tasks are moved, where appropriate, from highly qualified health workers to health workers with shorter training and fewer qualifications in order to make more efficient use of available resources for health" [23]. WHO has produced global guidelines and recommendations for task shifting, including a model where nurses initiate ART and doctors supervise and manage complex cases. In Uganda, Rwanda and Malawi, community health workers are now providing ART counselling and HIV testing [26].

The term 'task sharing' has been equated with task shifting [27]. We use the term here to denote staff who take on additional tasks without dropping (shifting) their pre-existing tasks. This paper compares district health facility workforce numbers, distribution and workload trends: the ratio of staff numbers to OPD visits, and to numbers of clients registered for ART, PMTCT and selected reproductive health programmes. Staff numbers and allocations to different activities for each year were obtained from health facility managers. If HIV/AIDS service delivery was becoming mainstreamed over the three year period reviewed, so that these services were becoming normalised at district health facilities, one would expect to see an increasing proportion of facility clinical staff delivering services such as ART and PMTCT.

## Methods

Two urban districts (Lusaka and Kabwe) and one rural district (Mumbwa) were purposefully selected because they were sufficiently close to Lusaka for significant HIV/AIDS service scale-up to be happening, which was likely to make heavy demands on health workers. At the time of the fieldwork in 2008, Lusaka and Kabwe were receiving funds to support HIV scale-up from PEPFAR and the Global Fund, whereas rural Mumbwa was only receiving funds from the latter. Lists of fixed health facilities providing HIV/AIDS services across the three districts were compiled with the support of District Health Management Teams.

Provision of ART services was the main criterion for inclusion in the study. All 29 facilities that were reported by district health teams as providing ART in 2007 were selected (24 government and 5 non-government/mission), excluding private for-profit and Ministry of Defence facilities. An additional 10 facilities were purposively selected so as to include non-ART providing facilities where significant scale-up of HIV services, such as PMTCT, VCT and AIDS home-based care, had started: 1 in Lusaka, 3 in Kabwe and 6 in Mumbwa. All district, mission and central hospitals were surveyed. Table 2 shows the types of HIV and non-HIV services provided across the three districts. All 39 sampled facilities provided VCT and malaria treatment; and 37 provided PMTCT and TB treatment. Most provided antenatal care (35), deliveries (28) and minor surgery (25).

**Table 2:** HIV and non-HIV services provided by sampled facilities (June 2008)

Service	All (39)	Lusaka (16)	Kabwe (10)	Mumbwa (13)	Urban (26)	Rural (13)	District (6)	Sub-district (33)
**Core HIV Services**								

ART	29	15	7	7	22	5	5	24

VCT	39	16	10	13	26	13	6	33

PMTCT	37	14	10	13	24	13	5	32

**HIV Support Services**								

Food/nutrition support	26	16	6	4	22	4	6	20

Income Generating Activities	13	7	2	4	9	4	2	11

Fee exemptions	36	16	10	10	26	10	5	31

Information and Education materials	38	16	9	13	25	13	6	32

Home Based Care	30	12	8	10	20	10	4	26

Spiritual support	23	9	5	9	14	9	2	21

**Non-HIV Services**								

Delivery (normal)	28	10	6	12	16	12	5	23

Antenatal care	35	13	9	13	22	13	5	30

TB test	24	14	8	2	22	2	6	18

TB treatment	37	16	8	13	24	13	6	31

Malaria test	37	14	10	13	24	13	6	31

Malaria treatment	39	16	10	13	26	13	6	33

Minor surgery	25	8	7	10	15	10	5	20

Major surgery	3	0	2	1	2	1	3	0

Health facility records (n = 39 facilities) were reviewed and data from the relevant facility departments were recorded on proformas (Additional file 1), quantifying service episodes and patient/client attendances between 2005 and 2007. Where facility records were missing or incomplete, electronic summaries were obtained from district health offices, which also supplied catchment population estimates, and we adjusted for earlier years. The denominator (number of facilities) for findings is usually less than 39 as not every facility surveyed was delivering specific services or because of non-reporting or missing data. Structured questionnaires (n = 39) were administered to health facility managers (Additional file 2) to ascertain types of services provided (including HIV and non-HIV priority services); categories and numbers of staff for the three years, and staff allocations to HIV services (Additional file 3). All instruments used in the 2008 surveys had been substantially modified and shortened based on lessons learned from a 2007 survey and were pre-tested again in 2008. Field workers received one week's training and were supervised by senior researchers during data collection.

Completed questionnaires and proformas were checked and data were double-entered on to EpiData v3.1 software, using validation checks and data entry restrictions. Data were exported to SPSS for cleaning and analysis. SAS v9.1 was used to merge datasets, generate queries of greater complexity and perform trend analyses, using the facility as the unit of analysis. Missing data were recorded as 'not applicable' where the facility was not designated for delivering that service; and recorded as 'not available' where there was an interruption in reported data for numbers of patients receiving a particular service. In both cases, the facility was excluded from trend analyses. This ensured that comparisons were based on consistent data across time periods and resulted in reduced numbers of facilities included in some trend analyses. Trend analyses were carried out to compare staff numbers with numbers of patients/clients registered for the principle HIV services (ART and PMTCT), non-HIV reproductive health services, and OPD visits within the same facilities. In other words, these were intra-facility analyses.

HIV versus non-HIV service utilisation trends were compared across the three districts (two urban and one rural), and also by level of facility (district and provincial hospitals versus health centres) and between ART versus non-ART providing facilities. Higher proportions of facilities had missing data for specific, common non-HIV clinical services, for example TB and malaria treatment; and these are consequently omitted from the findings. The results presented include trends for three consecutive years (2005-07). A methodological limitation was the reduction in sample sizes (numbers of facilities retained in the analyses), where there were missing data for one of these years, the consequences of which are considered in the Results and Discussion sections later. Medians with quartiles around the median ratios rather than means are used, as the latter give greater weight to facilities with large numbers of service users, which can swamp the effects seen in smaller facilities. Where medians and means showed different trends, this is presented and discussed in the text. Ethics approval for the study was granted by the University of Zambia Research Ethics Committee.

## Results

**Figure 1:**
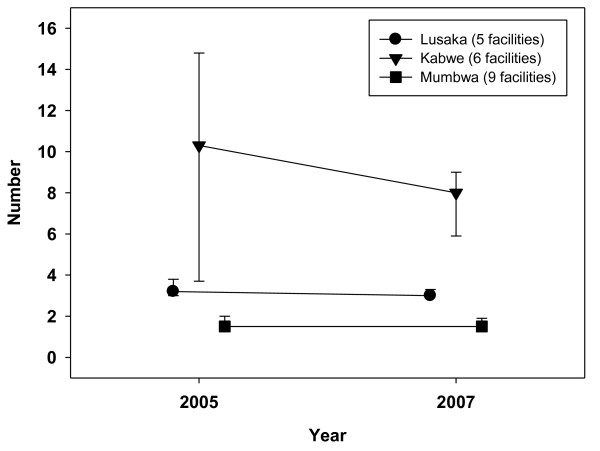
**Number of nurses per 10,000 catchment population, 2005-2007**.

**Figure 2:**
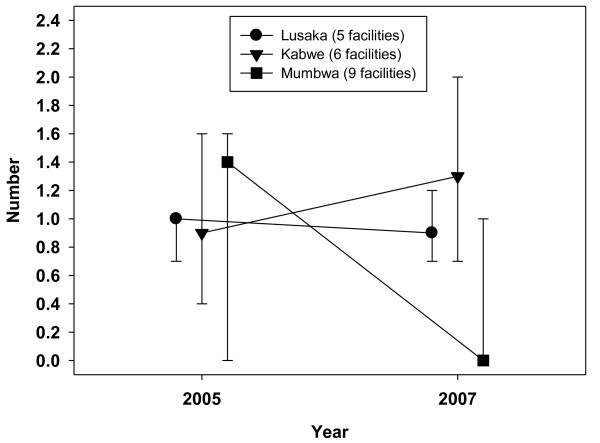
**Number of doctors and clinical officers per 10,000 catchment population, 2005-2007**.

Table 3 shows trends from 2005 to 2007 inclusive in the main categories of health workers in up to 30 facilities that provided complete data on staff complements for these three years. There was little change in Lusaka (the capital) and a slight decrease in the number of doctors, nurses/nurse midwives and clinical officers in the surveyed facilities in urban Kabwe and rural Mumbwa. There was a corresponding small increase in laboratory, pharmacy and records department staff and a somewhat larger rise in trained HIV counsellors in all three districts. Figure 1 shows the median ratios of nurses and Figure 2 the median ratios of doctors and clinical officers to population size in 20 facilities across the three districts. In other words, these were staff densities, using catchment population estimates recorded by district health offices as the denominators. The seven largest facilities, consisting of provincial, district and mission hospitals in Kabwe and Mumbwa, and some large non-government organisation (NGO) facilities providing ART in Lusaka, were excluded from the analysis because they lacked designated catchment populations.

**Table 3:** Numbers and population density of health workers by category and district, 2005 and 2007.

	Lusaka (n = 9)		Kabwe (n = 9)		Mumbwa (n = 12)		Total (n = 30)	
**Staff numbers & density**	**2005**	**2007**	**2005**	**2007**	**2005**	**2007**	**2005**	**2007**

Doctors	18	16	7	9	5*	6*	30*	31*

Nurses	160	166	260	240	68	65	***488***	***471***

Clinical officers	41	41	26	21	25	20	***92***	***82***

***Total clinical staff numbers: doctors, clinical officers and nurses***	***219***	***223***	***293***	***270***	***98***	***91***	***610***	***584***

Clinical staff density: number per 10 000 population ^§^	4.4	4.4	9.2	7.3	2.5	2.2	***4.9***	***4.6***

Laboratory technicians	10*	14*	23	19	3*	5*	***36***^***+***^	***38***^***+***^

Pharmacy technicians	18*	21*	10	11	4*	6*	***32***^***+***^	***38***^***+***^

Dedicated HIV counsellors	45	50	12*	22*	14*	21*	***71***^***+***^	**93**^**+**^

Records/registry clerks	38	42	16	18	2*	3*	56*	***63***^***+***^

***Total***	***330***	***350***	***354***	***340***	***121***	***126***	***805***	816^*^

* represents 1 facility short of the total number of facilities

+ represents 2 facilities short of the total number of facilities

^§ ^20 facilities: Lusaka = 5 facilities, Kabwe = 6 facilities, Mumbwa = 9 facilities. Other facilities lacked catchment populations

**Figure 3:**
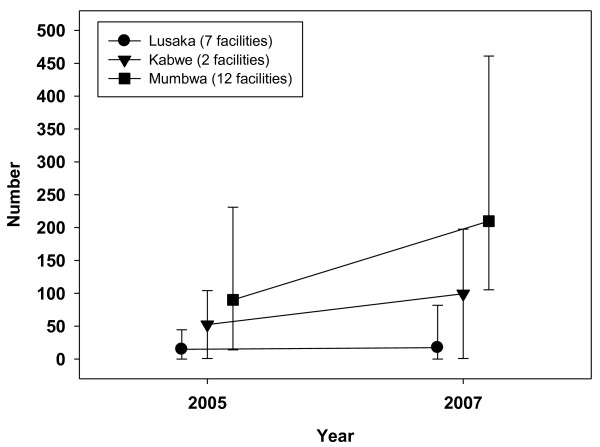
**Ratio of family planning clients to nurses, 2005-2007**.

**Figure 4:**
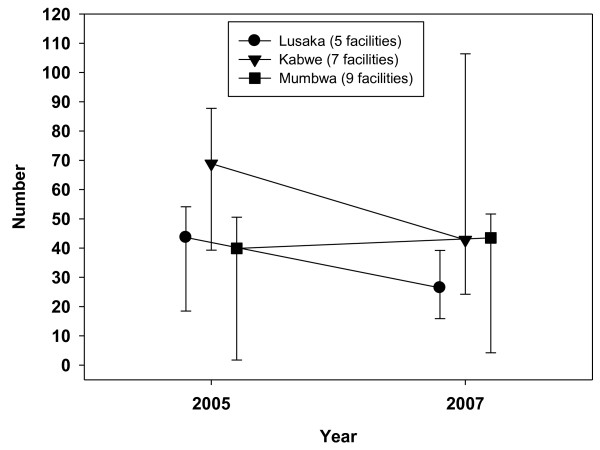
**Ratio of Outpatient Department clients per doctor and clinical officer per day, 2005-2007**.

Kabwe had the highest nurse density (median number of nurses per 10,000 catchment population) in 2005, at 10.3, which fell to 8.0 by 2007 (Figure 1). In Lusaka, nurse density was stable - the median fell slightly from 3.2 to 3.0, while the mean rose from 3.8 to 4.3. Rural Mumbwa had the lowest nurse density, at 1.5 (median and mean) in 2005 and 2007. Combined doctor and clinical officer densities were low, falling from a median of 1.4 to 0 in Mumbwa (from a mean of 1.0 to 0.4) between 2005 and 2007. Median densities were stable in Lusaka (around 1.0) and rose slightly in Kabwe from 0.9 to 1.3. Combined clinical staff densities (doctors, nurses and clinical officers) across the three districts were 3.4 per 10,000 in 2005, falling to 3.1 by the end of 2007. Mumbwa had lower clinical staff densities, falling from 2.7 to 2.1 over the three years. Figures 3 and 4 illustrate trends in workload for non-HIV services by designating specific major services to particular staff categories that would be expected to deliver these services: family planning to nurses and outpatient care to clinical officers and doctors. Such staff would also have been involved in the provision of other clinical services not included in this analysis, notably inpatient care. A within-facility analysis was conducted so as to illustrate effects at the facility level (median ratios with upper and lower quartiles).

The numbers of women who registered for family planning showed a slight fall from 37,093 (2005) to 33,653 (2007) in 29 reporting facilities. In 2005, family planning annual workload was highest in Mumbwa, rising from a median of 90 to 210 clients per nurse in 2007. This compared with a rise from 52 to 99 in Kabwe and much lower staff: client ratios in Lusaka (from 15 to 17). The difference in rural: urban family planning workload, which was already higher in rural Mumbwa in 2005, increased from six to twelve-fold over the three years. The numbers of women aged 15-49 who registered at antenatal clinics rose from 41,798 in 2005 to 46,656 in 2007 across all 29 facilities that reported completed data. Rural Mumbwa also had the highest antenatal clinic workload, which fell slightly from 435 in 2005 to 413 in 2007. It increased in Kabwe from 88 to 191 and remained lowest in Lusaka (falling from 63 to 58). Figure 4 shows trends in the median daily ambulatory outpatient visits per doctor and clinical officer in 21 facilities between 2005 and 2007. The nine facilities in rural Mumbwa experienced a slight increase from 40 to 44 median number of daily visits per clinical staff member; however, 25^th ^centiles remained the same and mean daily visits fell slightly from 33 to 31. In the seven facilities in Kabwe, median daily outpatient workload fell from 69 to 35, while mean workloads fell more modestly from 67 to 55. In the five facilities in Lusaka, median daily workload fell from 44 to 26, while mean workloads also fell more modestly (from 39 to 33).

A consequence of the small sample of facilities was the disproportionate effect of large changes in numbers of visits and workload in one Lusaka facility, which experienced a 50% fall in OPD visits and workload across the three years. Excluding it from the analysis resulted in only a small reduction of median daily workload from 31 (2005) to 28 (2007), and of mean workload from 35 to 34. Overall, across the three districts, median daily workload fell from 47 to 35 clients and mean workload fell slightly from 46 to 41. OPD numbers across the three districts also fell slightly from 397,374 in 2005 to 342,279 in 2007.

The numbers of clients on ART increased from 21,267 in 2005 to 44,311 in 2007 in all 24 facilities that reported the provision of ART across the three years. The numbers of ANC attendees who received an HIV test as part of a PMTCT programme increased from 19,939 in 2005 to 26,644 in 2007, in 16 reporting facilities, whilst those receiving antiretroviral PMTCT rose by more than half, from 4,303 to 6,797 in 15 facilities. The numbers of clients registered for ART in 16 facilities reporting complete human resource *and *ART record data rose from 13,113 (2005) to 22,811 (2007). Most ART scale-up was taking place in Lusaka, mainly in the University Teaching Hospital and four faith-based facilities. In 2005 Lusaka accounted for 95% of ART clients across the three districts, falling to 90% in 2007. Table 4 summarises data and trends in numbers of health workers delivering HIV-related services, 2005-07. Facility managers were asked if frontline staff (doctors, nurses, clinical officers and counsellors) were dedicated to specific HIV services, or were providing these in addition to other services, i.e. task sharing.

**Table 4:** Numbers of frontline health workers allocated to HIV related services, 2005 and 2007^1^

	Lusaka			Kabwe			Mumbwa			Total		
**Worker**	N *	2005**	2007**	N*	2005**	2007**	N*	2005**	2007**	N*	2005**	2007**

ART alone	6	45	45	1	0	4	0	0	0	7	45	49

ART and non-ART services	10	84	82	7	88	126	8	34	43	25	206	251

***Total ART***^***§***^	***10***	***129***	***127***	***7***	***88***	***130***	***8***	***34***	***43***	***25***	***251***	***300***

PMTCT alone	5	18	16	1	4	6	1	2	4	7	24	26

PMTCT and other services	7	65	72	9	58	84	11	22	32	27	145	188

***Total PMTCT***^***§***^	***8***	***83***	***88***	***9***	***62***	***90***	***11***	***24***	***36***	***28***	***169***	***214***

^1^Health workers include doctors, nurses, clinical officers and HIV counsellors

* n = numbers of facilities

** = numbers of health workers

^§ ^= total numbers of facilities is based on categories which are not mutually exclusive, ie a facility may have workers devoted to HIV services alone *and *workers providing HIV and other services

In rural Mumbwa, all staff involved in delivering ART were also delivering other non-HIV related services and the overall number contributing to ART provision rose by 26%, from 34 (2005) to 43 (2007). There was a 50% increase in the numbers of staff allocated to PMTCT in Mumbwa, 2005-07, 69% of whom were delivering other services. Kabwe had a similar pattern, with a similar 45% rise in staff delivering PMTCT and a 48% rise in numbers of staff delivering ART. By 2007, four staff members in Kabwe were dedicated to ART alone. Only in Lusaka, where the numbers of staff delivering ART and PMTCT changed little over the three years, was it common to find dedicated, standalone ART staff (45 at six of the ten facilities) in 2005 and 2007.

**Figure 5:**
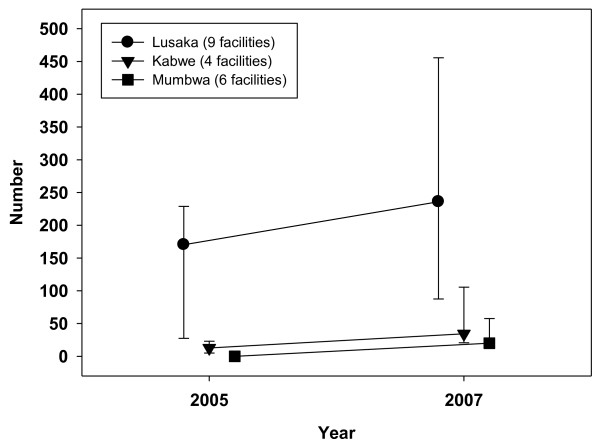
**Ratio of ART clients per ART worker, 2005-2007**.

**Figure 6:**
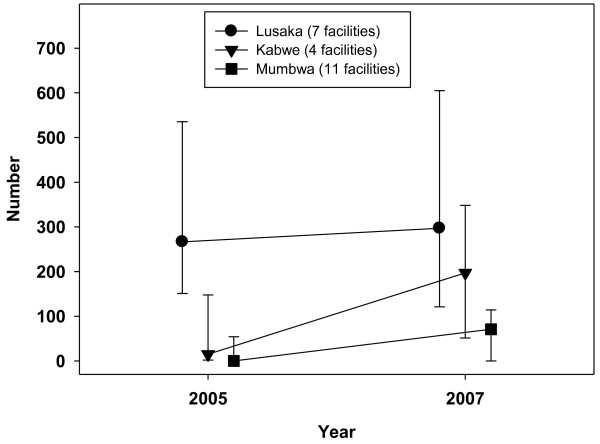
**Ratio of new ANC clients per PMTCT worker, 2005-2007**.

Figures 5 and 6 illustrate HIV workload ratios - median numbers of ART clients per designated ART health worker, and numbers of new antenatal clinic registrants per PMTCT worker. In contrast to non-HIV services, median ART workload (Figure 5) was much higher in Lusaka in 2005 and continued to rise, from 170 to 236 clients per ART worker (mean ART workload doubled from 162 to 322). In Kabwe, median ART workload almost doubled from a low base of 13 to 25, with mean workload increasing four-fold (from 14 to 63). In Mumbwa, median workload rose from zero to 18 (mean from 10 to 38) ART clients. PMTCT showed a similar pattern to ART, with much higher but only slowly rising workloads in Lusaka, a faster rise from a lower base in Mumbwa and a yet faster increase in workload in Kabwe (Figure 6), which also had the highest numbers of nurses per catchment population. Median numbers of HIV tests for laboratory technicians as part of VCT was heaviest in six facilities in Lusaka, where it increased gradually from 1131 clients (2005) to 1657 per lab technician (2007). In Kabwe, the median VCT client to laboratory staff ratio increased from 19 (2005) to 331 (2007) and Mumbwa experienced the largest increase from zero in 11 rural facilities in 2005, when VCT had not yet started, to 585 per lab technician in 2007.

Median ratios of ART staff to frontline health service providers (doctors, clinical officers, nurses and HIV counsellors) between 2005 and 2007, as reported by facility managers, are shown in Table 5. Ratios of PMTCT staff to frontline service providers are in Table 6. The within facility analysis shows that, in 2007, the highest proportion of frontline staff involved in ART delivery (task sharing) was in rural Mumbwa at 0.73 (73%). This compared to 0.44 in Kabwe and 0.50 in Lusaka. The numbers of staff contributing to ART delivery across the 21 facilities rose by 23% (from 209 to 258, with most of the increase occurring in Kabwe), between 2005 and 2007, whereas the total number of these frontline staff decreased slightly from 532 to 522.

**Table 5:** Staff numbers allocated to ART delivery as a ratio of all clinical staff, 2005 and 2007^1^.

District	Year	Numbers of facilities	Lower quartile	Median	Upper Quartile	Staff delivering ART	All clinical staff	Ratio
Lusaka	2005	6	0.40	0.53	0.82	87	155	0.56

	2007	6	0.40	0.50	0.80	81	153	0.53

Kabwe	2005	7	0.20	0.28	0.60	88	286	0.31

	2007	7	0.27	0.44	1.00	133	270	0.49

Mumbwa	2005	8	0.04	0.75	1.25	34	91	0.37

	2007	8	0.42	0.73	2.00	44	99	0.44

Total	2005	21	0.20	0.46	0.83	209	532	0.39

	2007	21	0.31	0.59	1.00	258	522	0.49

^1^Clinical staff include doctors, nurses, clinical officers and HIV counsellors

**Table 6:** Staff numbers allocated to PMTCT delivery as a ratio of all clinical staff, 2005 and 2007^1^

District	Year	Numbers of facilities	Lower quartile	Median	Upper Quartile	Staff delivering PMTCT	All clinical staff	Ratio
Lusaka	2005	5	0.24	0.36	0.39	56	168	0.33

	2007	5	0.27	0.34	0.43	64	185	0.35

Kabwe	2005	7	0.05	0.20	0.60	53	241	0.22

	2007	7	0.42	0.81	1.00	79	224	0.35

Mumbwa	2005	11	0.00	0.25	0.50	24	110	0.22

	2007	11	0.31	0.60	2.00	36	113	0.32

Total	2005	23	0.05	0.25	0.50	133	519	0.26

	2007	23	0.31	0.58	1.00	179	522	0.34

^1^Clinical staff include doctors, nurses, clinical officers and HIV counsellors

The trend for PMTCT across 23 facilities (Table 6) was slightly different, although showing a similar contrast between Lusaka and the other two districts. By 2007, there had been a four-fold increase in PMTCT task sharing in Kabwe, where staff allocated to PMTCT as a ratio of all frontline clinical staff rose from 0.20 to 0.81, and a two-three fold increase in Mumbwa, from 0.25 to 0.60. There was little change in Lusaka with a stable proportion (around one third) of frontline staff involved in PMTCT delivery. By 2007, the numbers of staff contributing to PMTCT delivery had increased by 35%, with virtually no change in the numbers of staff one would expect to be required to deliver these services, as well as other routine clinical services (see Table 3).

## Discussion

Facility level findings confirm and quantify reports from recent qualitative studies that new funds for HIV are increasing the strain on Zambian health workers through the resultant demands on them to deliver more services to more people [12,13]. Findings also quantify a bigger and growing impact on staff in rural facilities [8,28]. Firstly, the study revealed staff densities that were between one third and a half of those reported nationally; and much lower densities, especially in the surveyed rural district (Mumbwa), than the recommended minimum workforce density needed to provide essential health services. Countries with an average of fewer than 2.5 doctors/nurses and midwives per 1,000 population (25 per 10,000) failed to achieve an 80% coverage rate for deliveries by skilled birth attendants or for measles immunisations [1]. In 2004, Zambia's ratio was reported to be one third of this norm, at 7.9 per 10,000 people. By 2007, staff density had reportedly risen to only 9.8 per 10,000 [29], despite an additional $900 million funding from PEPFAR and the Global Fund to support HIV/AIDS service scale-up [30-32].

Our findings, from three districts, showed clinical staff densities less than half of those stated in the Ministry of Health 2007 review, using a measure that combined doctors, nurses and clinical officers. Low nurse densities (Figure 1), which fell from 2.8 to 2.6 across the three districts, also calls into question a reported density in Zambia of 17.4 nurses and 2.7 midwives per 10,000 in the 2006 WHO report [1]. Rural Mumbwa, where the clinical staff density fell from 2.7 to 2.1 per 10,000 population, was representative of districts categorised as 'poor infrastructure rural' in the Global Fund Five Year Evaluation [8], which reported a 2007 average staff density of 2.6 in three of six rural Zambian districts studied.

Secondly, this study utilised routine health service data to unpack, locate and quantify workload levels and trends. Workload was the most important factor contributing to a recently reported occupational burnout rate of 51% in urban health workers in Zambia [21]. In our study, low and falling nurse/nurse-midwife numbers were contributing to high and often rising antenatal and family planning clinic workload ratios, especially in rural Mumbwa. Scale-up of PMTCT services occurred rapidly in Zambia between 2005 and 2007. Nationally, the numbers of sites delivering PMTCT increased from 67 in 2005 to 678 in 2007, and coverage of pregnant women needing and receiving ART had increased by almost one third from 29.7% in 2006 to 39.1% by 2007 (Table 1). In the surveyed facilities in our study, the numbers of clients registered for PMTCT also increased rapidly across the three years, 2005 to 2007, and there was a slight increase in the number of women registered for antenatal care and a slight decrease in the numbers registered for family planning services.

OPD workload, which was attributed in the analysis to doctors and clinical officers, showed a marked reduction in the median ratio of daily visits per staff member in Lusaka and Kabwe from 2005 to 2007. Workload reductions were more modest when using mean daily outpatient visits as the workload measure. There was a slight increase in Mumbwa, where median outpatient workload went from lowest to highest of the three districts between 2005 and 2007. This, together with the high and/or rising antenatal clinic and family planning workloads, support the reports that the strain on health workers is growing faster in rural areas. The fall in OPD clients suggests the possibility of some crowding out of non-HIV services. We attempt to answer the 'crowding' question - was the increase in HIV related workload crowding out, or was it associated with an increase in, the number of clients receiving population maternal and child health services? - in another paper [33].

There are some important caveats with respect to the workload estimates: first is the small sample sizes (especially when stratifying by district), which meant that variations in outpatient visit numbers in one or two facilities had large effects. Second is that other studies have measured workload more precisely, using activity time of staff [17,19]. Thirdly, in calculating daily workload, we assumed that there were 250 working days in the year, taking into account national holidays. This is likely to have under-estimated real daily workloads, in that staff were likely to have been absent from their facilities for an unmeasured number of days for a variety of reasons including out-of-facility workshops, sick leave and funerals. It is also likely that clinical staff, especially doctors, will have divided their time between the OPD and inpatient wards. Fourthly, a more precise and validated attribution of types of services to different types of staff might have produced different workload ratios.

However, the value of these intra-facility analyses is that they demonstrate the correlation of client/patient numbers with staff numbers within each facility, not just aggregated at a district level. Also, the use of median and mean measures showed that routine outpatient workload fell only slightly and was at or above a workload estimate of 35 visits per health worker per day, assuming that this would be a reasonable daily workload for frontline health workers. Despite the considerable attention given to workload measures, we found little discussion of the relative merits of using means and medians. The latter, especially when quartiles or other centiles are included, reduce the effects of changes in very large facilities with large numbers (and variations) of patients/clients and give a measure of workload that take into account effects in small as well as large facilities. In small sample surveys, there are advantages in using both measures.

The prediction by Kombe et al [34] that facilities might not scale-up HIV services due to lack of staff was not borne out in this study. Findings (Figures 5 and 6), based on HIV and non-HIV service data collected from health facilities, show similar upward trends in HIV-related client numbers and workload across rural and urban facilities, with much higher ART and PMTCT workload already evident in Lusaka by 2005. The latter can be attributed to higher demand in the capital city, where the HIV prevalence was higher, and to increasing specialisation in urban facilities with standalone ART and PMTCT workers. Tables 4 to 6 show large increases in the percentages of frontline staff allocated to ART and PMTCT delivery between 2005 and 2007. This was not only in rural Mumbwa, where almost three quarters of staff were supporting ART, but also in Kabwe where a within-facility analysis revealed a more dramatic increase in additional duties: the average percentage of frontline staff supporting PMTCT rose from 20% to 81%. In both districts, the absolute numbers of clinical and nursing staff in surveyed facilities *decreased *over the three years period. A plausible explanation is that an increasing proportion of clinical and nursing staff, whose numbers were static, had taken on ART and PMTCT delivery, on top of their other non-HIV duties.

The term 'task shifting' has been widely discussed [3,4,35] and the related term 'task sharing' has sometimes been assumed (incorrectly in our view) to be synonymous with task shifting [27]. It is likely that services that require the expertise of clinical staff, such as starting patients on ART and PMTCT, require existing clinical staff to share and pick up the additional workload, i.e. task sharing. Recent guidelines from the National HIV/AIDS Council of Zambia (NAC) and the Ministry of Health [36] state that only doctors and clinical officers are legally recognised to prescribe ART in Zambia. However, WHO task shifting guidelines (2008) recommend that nurses should also be responsible for initiating and prescribing ART [23].

Over time, as more clinical staff were trained and standardised protocols and guidelines were introduced, the probable explanation for increasing ratios of clinical staff to ART and PMTCT in our study is that HIV/AIDS services were increasingly mainstreamed and therefore normalised and integrated into normal facility staff workloads. From the perspective of access and availability of services and the institutionalisation of HIV/AIDS control at the facility level, this can be seen as a positive development. However, this paper takes the perspective of workload and the implications of HIV service scale up on health workers. It should be noted, also, that the data on task sharing that were obtained from health facility managers, which showed increasing numbers of staff allocated to the provision of HIV services, 2005-2007, did not include a measurement of what proportion of time staff spent on HIV and non-HIV services.

A study which assessed the role of lay counsellors in Zambia, found that they provided up to 70% of HIV counselling and testing services at health facilities [37]. We did not quantify the number and trends in lay or voluntary counsellors, 2005-07, and the staff designated as counsellors were trained staff employed by these facilities. It is possible that some facility managers designated staff as ART and PMTCT providers, who were not part of their formal staff complement, in the responses to the survey. It is also likely that practices in a small clinic employing a clinical officer and one or two nurses, who take on a range of service activities, is different to a district hospital or large urban clinic where more specialisation occurs. Therefore quantifying an increased (and by 2007 quite high) proportion of existing frontline staff as taking on these responsibilities on top of their other non-HIV workload is still a hypothesis. This requires testing with different data collection tools, or better still, improved routine information systems. However, staff shortages and rapidly increasing HIV workload were a reality, as also demonstrated in increases in the numbers of HIV laboratory tests. In Mumbwa, where a combination of HIV and non-HIV workload increases were greatest, eight surveyed rural health centres delivering routine services and increasingly delivering HIV services were staffed with an average of one nurse and one clinical officer each, which is consistent with national reports of greater staff shortages in rural areas [2].

The Government of Zambia has recognised that the health sector is facing a human resource crisis and that there are shortages of health workers at every level of the system [2]. There is currently no single source or database of employees working in government and mission (faith-based) hospitals and health centres, although multiple sources of data exist. Lack of data quantifying staff distribution and workload may have contributed to the lack of follow-through on staffing recommendations to meet the anticipated HIV service challenges [22,38]. National policy makers are aware of the underlying problems, for example: "placements of staff ... favour urban areas at the expense of rural areas" [29]. The causes have also been well documented and responses to lack of housing and adequate living conditions were incorporated into the 2003 Zambia Health Worker Rural Retention Scheme [39]. This scheme aimed to decrease attrition rates in rural districts by providing a monthly stipend, housing rehabilitation, vehicle loans and facility incentives. In return, the health worker is required to work in a rural area for three years [2,39]. The scheme's limited success has been attributed to a lack of accommodation, short timeframe for retention allowances, and eligibility criteria, which until 2007 only included doctors [29].

According to Zambia's Human Resources for Health Strategic Plan 2006-2010, the cost of achieving the needed establishment of health workers would increase from Kwacha 23 billion to Kwacha 651 billion; and in order to achieve this, even in a phased manner, staffing levels in other sectors would have to fall [2]. The human resource crisis has been compounded by the fact that the Ministry of Health (due to budgetary ceilings) has had fixed staff establishments for all districts and health facilities. By 2008, 23% of Global Fund resources had been committed to human resources [40] but not to hire or to train new health workers. The focus has been on in-service training and workshops to improve the capacity of existing staff. PEPFAR had also not funded basic training or the hiring of new health workers up to 2007; although as part of its reauthorisation, it was announced in August 2008 that PEPFAR would support the training of at least 140,000 new healthcare workers in HIV/AIDS prevention and care [41].

## Conclusion

This paper has aimed to demonstrate the potential of intra-facility analyses of routine health worker data for identifying and measuring where health worker crises are most acute, or developing, and where strategic human resource allocation responses are needed. Zambia's 2012 target is 100% reporting of a human resource sub-system within an integrated health information system [42]. This will be of little value if data are not transformed into information, and then explained and used. The strengthening of capacity to collect, validate, analyse and use data will require an information cultural transformation at all levels, as recognised in the 2009 Health Information Systems Strategic Plan [42,43]. The findings in this study give some indication of additional and growing workload on staff, due to rapid scale-up of HIV services superimposed on routine care, without an increase in the numbers of formally trained health workers. While workshops and in-service training of existing health workers, task shifting and incorporating lay or volunteer workers into HIV and other disease control efforts are essential to scaling up HIV/AIDS services, on their own they will not solve the health worker shortage crisis. We did not capture the extent of task shifting to lay counsellors. However, others have questioned whether clinical services (HIV and non-HIV) can be delivered safely and effectively without a sufficient complement of clinical staff (doctors, nurses and clinical officers) [44].

Much of the recent focus of human resources for health research has been on task shifting. Reviews of the evidence have shown that task shifting 'can lead to improvements in access, coverage and quality of health services at a comparable or lower cost than traditional delivery models' [4,35]. Others have argued that task shifting should not be considered a panacea [44]. Our analysis suggests that task sharing as well as task shifting is occurring, i.e. that the few available staff are taking on additional work, especially in small rural health facilities. Further research is needed that measures types of work and workload across the spectrum of staff working at the health facility level, including trained and lay (or volunteer) staff. This study did not measure total facility workload, taking into account HIV and all non-HIV services, including inpatient as well as outpatient care. Further studies are needed on larger representative samples to ascertain if similar HIV and non-HIV workload trends are occurring more widely. Health managers need to be able to measure workload routinely across HIV and non-HIV services, and ensure that staff are allocated fairly and appropriately. The value as well as the limitation of this study is that it attempted to use and analyse routine data to measure health worker effects. Future work should proceed to the next stage, in the form of sequential mixed methods explanatory studies, where qualitative methods are used to explore and explain quantitative trends and quality of care issues are explored in more detail [18].

Dreesch and colleagues [18] have proposed methods for estimating staff requirements for priority health interventions, which recognise the effects of shared skills, task sharing between health professionals and multiple tasks performed by particular health workers. Daviaud and Chopra [17] working in South Africa, have further developed the WHO Workload Indictors of Staffing Need (WISN) model. This includes the allocation of types of consultation to categories of health workers, length of consultation and management time, clinical support requirements and time off duty amongst other indicators. Our analyses focused on the much smaller and simpler set of indicators that can be measured using routine health information currently produced in the less sophisticated health information system of Zambia.

This study, as well as throwing light on the health worker crisis at the health facility level, is one of the first attempts to quantify the effects of GHIs on district level services. Persistent efforts were made by the research team to track and document sources and amounts of funds disbursed to and spent at district and facility levels; however this proved impossible. This meant that attribution of health worker and other health systems effects to specific GHIs was not possible. Other recent studies have also found similar difficulties [12]. More important than attribution is the need for a collective response - by the government of Zambia, the GHIs and other donors - whereby they will prioritise support to increasing formal basic health worker training, recruitment and retention. To support these priorities, an effective, functioning human resource information system needs to be in place, which can be used to monitor staff distribution, client/patient numbers and thereby staff workload, and inform implementation of the Human Resources for Health Strategic Plan. Routine health information exists in most stable settings, and the Zambian health information system used in this study (albeit flawed) has helped to demonstrate that the human resources crisis is worsening, especially in a rural district of Zambia. Policy-makers require reliable, timely and regular evidence to monitor the effects of their decisions and they should not be reliant on the occasional studies produced by health services researchers.

## Competing interests

The authors declare that they have no competing interests.

## Authors' contributions

AW participated in data collection, data analysis and interpretation, and drafting of the article. PN participated in study design, data collection, data analysis and interpretation, and drafting of the article. JS participated in data collection, data analysis and interpretation, and drafting of the article. PD participated in data analysis and interpretation, and drafting of the article. RB participated in study design, data analysis and interpretation, and drafting of the article. All authors have seen and approved the final version.

## Pre-publication history

The pre-publication history for this paper can be accessed here:

http://www.biomedcentral.com/1472-6963/10/272/prepub

## Supplementary Material

Additional file 1 **Health Facility Records/Register Review**. A *proforma *used to quantify service episodes and patient/client attendances (HIV and non-HIV) between 2005 and 2007. Click here for file

Additional file 2 **Health Facility Survey with Health Facility Level Manager/NGO Manager**. A structured questionnaire, administered to health facility managers, to ascertain types of services (HIV and non-HIV) provided within each facility. Click here for file

Additional file 3 **Health Facility Survey - Human Resources**. A structured questionnaire, administered to health facility managers, to ascertain health worker categories, trends over time and staff allocations to services. Click here for file
