# Supplementary material for: Task sharing in Zambia: HIV service scale-up compounds the human resource crisis
Source: BMC Health Serv Res. 2010 Sep 17;10:272. doi: 10.1186/1472-6963-10-272 (PMC2955013; doi:10.1186/1472-6963-10-272)
Supplement: Additional file 1 — Health Facility Records/Register Review. A proforma used to quantify service episodes and patient/client attendances (HIV and non-HIV) between 2005 and 2007. [file 1472-6963-10-272-S1.DOC]

Global HIV/AIDS Initiatives in Zambia

Health Facility Records/Register Review

Phase 2 - June 2008

Section 1 Outpatient services

Section 2 VCT services

Section 3 ART services

Section 4 PMTCT services

### Questionnaire No.: [________] *Office use only* Date of Interview (dd/mm/yyyy): [____/____/________] Name of Facility ____________________________­________

Location of Facility____________________________________

Result Code 1 Completed

2 Respondent not available

3 Refused [__]

4 Partially completed

5 Other

Specify____________________________

Research Interviewer (RI) [____]

RI Sign.___________ Checked by Supervisor_____________ Date___/___/_______

Interviewer: If there are gaps in the records, refer to the facility manager.

1. Outpatient services

| 101. Numbers of outpatient seen during 2004, 2005, 2006 and 2007 | | | | | |
| --- | --- | --- | --- | --- | --- |
|  | Service | Total 2004 | Total 2005 | Total 2006 | Total 2007 |
| A | Total Outpatients (visits) |  |  |  |  |
| B | Malaria (clients) |  |  |  |  |
| C | Malaria (visits) |  |  |  |  |
| D | ANC services (clients) |  |  |  |  |
| E | ANC services (visits) |  |  |  |  |
| F | No. of sputum smear positive TB cases diagnosed |  |  |  |  |
| G | No. of sputum smear positive TB cases who successfully completed treatment |  |  |  |  |
| H | Family Planning (clients) |  |  |  |  |
| I | Family Planning (visits) |  |  |  |  |
| J | Paediatric outpatient (clients) |  |  |  |  |
| K | Paediatric outpatient (visits) |  |  |  |  |
| L | Total measles vaccinations |  |  |  |  |

102. Outpatients 2007

|  |  | Jan07 | Feb 07 | Mar 07 | April 07 | M May 2007 | June07 | July 07 | Aug 07 | Sept 07 | Oct 07 | Nov 07 | Dec 07 |
| --- | --- | --- | --- | --- | --- | --- | --- | --- | --- | --- | --- | --- | --- |
| A | Total outpatients (visits) |  |  |  |  |  |  |  |  |  |  |  |  |
| B | Malaria (clients) |  |  |  |  |  |  |  |  |  |  |  |  |
| C | Malaria (visits) |  |  |  |  |  |  |  |  |  |  |  |  |
| D | ANC (clients) |  |  |  |  |  |  |  |  |  |  |  |  |
| E | ANC (visits) |  |  |  |  |  |  |  |  |  |  |  |  |
| F | No. of smear positive TB cases diagnosed |  |  |  |  |  |  |  |  |  |  |  |  |
| G | No. of smear positive cases who successfully completed treatment |  |  |  |  |  |  |  |  |  |  |  |  |
| H | Family planning (clients) |  |  |  |  |  |  |  |  |  |  |  |  |
| I | Family planning (visits) |  |  |  |  |  |  |  |  |  |  |  |  |
| J | Paediatric outpatient (clients) |  |  |  |  |  |  |  |  |  |  |  |  |
| K | Paediatric outpatient (visits) |  |  |  |  |  |  |  |  |  |  |  |  |
| L | Total measles vaccinations |  |  |  |  |  |  |  |  |  |  |  |  |

103. Outpatients 2008

|  |  | Jan08 | Feb 08 | Mar 08 | April 08 | May 08 |
| --- | --- | --- | --- | --- | --- | --- |
| A | Total outpatients (Clients) |  |  |  |  |  |
| B | Total outpatients (Visits) |  |  |  |  |  |
| C | Malaria (Clients) |  |  |  |  |  |
| D | Malaria (Visits) |  |  |  |  |  |
| E | ANC (Clients) |  |  |  |  |  |
| F | ANC (visits) |  |  |  |  |  |
| G | No. of smear positive TB cases diagnosed |  |  |  |  |  |
| H | No. of smear positive cases who successfully completed treatment |  |  |  |  |  |
| I | Family planning (Clients) |  |  |  |  |  |
| J | Family planning (Visits) |  |  |  |  |  |
| K | Paediatric outpatient (Clients) |  |  |  |  |  |
| L | Paediatric outpatient (Visits) |  |  |  |  |  |
| M | Total measles vaccinations |  |  |  |  |  |

2. VCT Services

| 2004 | | | | | | 2005 | | | | |
| --- | --- | --- | --- | --- | --- | --- | --- | --- | --- | --- |
|  | Total |  | Total | F | M | Total |  | Total | Male | Female |
| a. Persons receiving HIV counselling as part of VCT |  | Children |  |  |  |  | Children |  |  |  |
|  |  | Adults |  |  |  |  | Adults |  |  |  |
| b. Persons receiving HIV testing as part of VCT |  | Children |  |  |  |  | Children |  |  |  |
|  |  | Adults |  |  |  |  | Adults |  |  |  |
| c. Persons testing positive for HIV |  | Children |  |  |  |  | Children |  |  |  |
|  |  | Adults |  |  |  |  | Adults |  |  |  |

| 2006 | | | | | | 2007 | | | | |
| --- | --- | --- | --- | --- | --- | --- | --- | --- | --- | --- |
|  | Total |  | Total | F | M | Total |  | Total | Male | Female |
| a. Persons receiving HIV counselling as part of VCT |  | Children |  |  |  |  | Children |  |  |  |
|  |  | Adults |  |  |  |  | Adults |  |  |  |
| b. Persons receiving HIV testing as part of VCT |  | Children |  |  |  |  | Children |  |  |  |
|  |  | Adults |  |  |  |  | Adults |  |  |  |
| c. Persons testing positive for HIV |  | Children |  |  |  |  | Children |  |  |  |
|  |  | Adults |  |  |  |  | Adults |  |  |  |

105. VCT Services 2007

|  |  | Jan 07 | Feb 07 | Mar 07 | April 07 | May 07 | June 07 | July 07 | Aug 07 | Sept 07 | Oct 07 | Nov 07 | Dec 07 |
| --- | --- | --- | --- | --- | --- | --- | --- | --- | --- | --- | --- | --- | --- |
| Persons receiving HIV counselling as part of VCT | Total |  |  |  |  |  |  |  |  |  |  |  |  |
|  | Children |  |  |  |  |  |  |  |  |  |  |  |  |
|  | Adults |  |  |  |  |  |  |  |  |  |  |  |  |
| Persons receiving HIV testing as part of VCT | Total |  |  |  |  |  |  |  |  |  |  |  |  |
|  | Children |  |  |  |  |  |  |  |  |  |  |  |  |
|  | Adults |  |  |  |  |  |  |  |  |  |  |  |  |
| Persons testing positive for HIV | Total |  |  |  |  |  |  |  |  |  |  |  |  |
|  | Children |  |  |  |  |  |  |  |  |  |  |  |  |
|  | Adults |  |  |  |  |  |  |  |  |  |  |  |  |

106. VCT Services 2008

|  |  | Jan 08 | Feb 08 | Mar 08 | April 08 | May 08 |
| --- | --- | --- | --- | --- | --- | --- |
| Persons receiving HIV counselling as part of VCT | Children |  |  |  |  |  |
|  | Adults |  |  |  |  |  |
| Persons receiving HIV testing as part of VCT | Children |  |  |  |  |  |
|  | Adults |  |  |  |  |  |
| Persons testing positive for HIV | Children |  |  |  |  |  |
|  | Adults |  |  |  |  |  |

3. ART Services

107. How many clients received ART services in the facility?

|  |  | 2004 | 2005 | 2006 | 2007 |
| --- | --- | --- | --- | --- | --- |
| Totals | |  |  |  |  |
| Female | Children |  |  |  |  |
|  | Adults |  |  |  |  |
| Male | Children |  |  |  |  |
|  | Adults |  |  |  |  |

ART clients receiving treatment in 2007

| 108. How many clients received ART in 2007? | | | | | | | |  | | | | | | |
| --- | --- | --- | --- | --- | --- | --- | --- | --- | --- | --- | --- | --- | --- | --- |
|  | | | Jan  07 | Feb 07 | Mar 07 | April 07 | M May 0707 | June  07 | July07 | Aug 07 | Sept 07 | Oct  07 | Nov 07 | Dec 07 |
| ART (patients/ clients) | Totals | |  |  |  |  |  |  |  |  |  |  |  |  |
|  | Female | Child  ren |  |  |  |  |  |  |  |  |  |  |  |  |
|  |  | Adults |  |  |  |  |  |  |  |  |  |  |  |  |
|  | Male | Child  ren |  |  |  |  |  |  |  |  |  |  |  |  |
|  |  | Adults |  |  |  |  |  |  |  |  |  |  |  |  |

109. ART clients receiving treatment in 2008

|  |  | | Jan 08 | Feb 08 | Mar 08 | April 08 | M May 08 |
| --- | --- | --- | --- | --- | --- | --- | --- |
| ART (patients/clients) | Totals | |  |  |  |  |  |
|  | Female | Children |  |  |  |  |  |
|  |  | Adults |  |  |  |  |  |
|  | Male | Children |  |  |  |  |  |
|  |  | Adults |  |  |  |  |  |

4. PMTCT Services

110. PMTCT services

|  |  | 2004 | 2005 | 2006 | 2007 |
| --- | --- | --- | --- | --- | --- |
| A | No. of new ANC attendants |  |  |  |  |
| B | Total No. of ANC attendants receiving HIV testing |  |  |  |  |
| C | Total No. of ANC attendants testing positive |  |  |  |  |
| D | Total No. of HIV positive attendants receiving treatment (PMTCT) |  |  |  |  |

111. PMTCT services 2007

|  |  | Jan 07 | Feb 07 | Mar 07 | April 07 | May 07 | June 07 | July 07 | Aug 07 | Sept 07 | Oct 07 | Nov 07 | Dec 07 |
| --- | --- | --- | --- | --- | --- | --- | --- | --- | --- | --- | --- | --- | --- |
| A | No. of new ANC attendants. |  |  |  |  |  |  |  |  |  |  |  |  |
| B | Total No. of ANC attendants receiving HIV testing. |  |  |  |  |  |  |  |  |  |  |  |  |
| C | Total No. of ANC attendants testing positive |  |  |  |  |  |  |  |  |  |  |  |  |
| D | Total No. of HIV positive attendants receiving treatment |  |  |  |  |  |  |  |  |  |  |  |  |

112. PMTCT 2008

|  |  | Jan 08 | Feb 08 | Mar 08 | April 08 | M May 08 |
| --- | --- | --- | --- | --- | --- | --- |
| A | No. of new ANC attendees |  |  |  |  |  |
| B | Total No of ANC attendants receiving HIV test results |  |  |  |  |  |
| C | Total No. of ANC attendants testing positive |  |  |  |  |  |
| D | Total No. of HIV positive attendees receiving treatment |  |  |  |  |  |

5. Condom distribution

| 113. How many condoms were distributed from this facility between 2004 and 2008? | | | | | |
| --- | --- | --- | --- | --- | --- |
|  | Jan to Dec 2004 | Jan to Dec 2005 | Jan to Dec 2006 | Jan to Dec 2007 | Jan – May 2008 |
| Male Condoms |  |  |  |  |  |
| Female Condoms |  |  |  |  |  |
