# Supplementary material for: Task sharing in Zambia: HIV service scale-up compounds the human resource crisis
Source: BMC Health Serv Res. 2010 Sep 17;10:272. doi: 10.1186/1472-6963-10-272 (PMC2955013; doi:10.1186/1472-6963-10-272)
Supplement: Additional file 2 — Health Facility Survey with Health Facility Level Manager/NGO Manager. A structured questionnaire, administered to health facility managers, to ascertain types of services (HIV and non-HIV) provided within each facility. [file 1472-6963-10-272-S2.DOC]

Global HIV/AIDS Initiatives in Zambia

**Health Facility Survey - Facility Level Manager / NGO Manager**

**Phase 2 – June 2008**

# Instructions

- To be administered at the Health Facility
- Explain the purpose of the study and politely request respondent for permission to proceed as detailed in the Informed Consent Statement.
- Circle the code corresponding to the response and fill the code of the response in the space provided against each question.
- Particular attention should be paid to skips and interviewer instructions throughout the questionnaire.

### Questionnaire No.: [________] *Office use only* Date of Interview (dd/mm/yyyy): [____/____/________] Name of Facility ____________________________­________

Location of Facility ____________________________________

Type/level of Facility

**1** Provincial Hospital

**2** District Hospital

**3** Hospital

**4** Health Centre

**5** Health Post

**6** VCT Centre

**7** Fixed Outreach **[__]**

**8** Home-based care

**9** Other

Specify____________________________

Rural or urban facility **1** Urban

**2** Rural  **[__]**

Managing Authority **1** Government

**2** NGO/CBO

**3** FBO/Mission **[__]**

**4** Other

Specify____________________________

Result Code **1** Completed

**2** Respondent not available

**3** Refused **[__]**

**4** Partially completed

**5** Other

Specify____________________________

Research Interviewer (RI) **[____]**

**RI Sign.___________ Checked by Supervisor__________Date___/___/_____**

**Start time ____________**

| **Section 1: Facility / services information** | | | | |
| --- | --- | --- | --- | --- |
| 101 | Which Year and Month did you come to this facility | Year__________ Month________ |  |  |
| 102 | What is your professional qualification? | Medical Doctor (General) **01**  Medical Doctor (Specialist) **02**  Clinical Officer **03**  Registered Nurse (ZRN) **04**  Registered Nurse (ZRN) Midwife **05**  Enrolled Nurse (ZEN) **06**  Enrolled Nurse (ZEN) Midwife **07**  Pharmacist **08**  Pharmacy Technician **09**  Laboratory Technician **10**  Records/ Registry **11**  Other **66**  Specify **_______________________** | [____] |  |
| 103 | Are you personally involved in the provision of ART? | Yes **1**  No **2**  There is no ART at facility **3** | **[__]** |  |
| 104 | Do you have an estimate of the size of the catchment population that this facility serves, that is, the size of the population living in the area served by this hospital? | Yes **1**  No **2**  No catchment area **3**  Don’t know catchment population **4** | **[__]** | **If No, go to 106** |
| 105 | *IF YES*: what is the population size, that is how many people? | **> 50,000 1**  **10,000 – 49,999 2**  **1,000 – 9,999 3**  **100 – 999 4**  **< 100 5**  Also Record number:[_______________] | **[__]** |  |
| 106 | Does this facility provide the following services?  ***READ out and circle 1 or 2 from each option*** | | Yes=1; No=2 |  | | --- | --- | |  | Yes No | | a) Delivery (normal) | **1 2** | | b) Delivery (Caesarian Section) | **1 2** | | c) Antenatal Care (ANC) | **1 2** | | d) Tuberculosis Test | **1 2** | | e) Tuberculosis treatment | **1 2** | | f) Malaria test | **1 2** | | g) Malaria treatment | **1 2** | | h) Minor surgery | **1 2** | | i) Major surgery | **1 2** | | j) Other | **1 2** | | **Specify ______________________** | | | |  | | --- | |  | | [__] | | [__] | | [__] | | [__] | | [__] | | [__] | | [__] | | [__] | | [__] | |  |
| 107 | Does this facility provide **ART** services | Yes 1 No **2** | **[__]** |  |
| 108 | What year were ART services first provided in this facility? | Year: __________ |  |  |
| 109 | Does this facility provide **VCT** services? | Yes 1No 2 | **[__]** |  |
| 110 | What year were VCT services first provided in this facility? | Year: __________ |  |  |
| 111 | Does this facility provide **PMTCT** services? | Yes 1No 2 | **[__]** |  |
| 112 | When were PMTCT services first provided at this facility? | Year: ________ |  |  |
| 113 | Which of the following support services does this facility provide to people infected with HIV and their families?  ***(Read out the answers)*** | | Yes=1; No=2 |  | | --- | --- | |  | Yes No | | a) Counselling (for people who are HIV positive) | **1 2** | | b) Food/nutritional support | **1 2** | | c) Income generating projects | **1 2** | | d) Fee exemptions | **1 2** | | e) Support for schooling/education | **1 2** | | f) Information and Educational materials (contraception, HIV/AIDS, STIs, nutrition, pregnancy) | **1 2** | | g) Home Based Care | **1 2** | | h) Clothing | **1 2** | | i) Support to families of people living with HIV/AIDS | **1 2** | | j) Support to widows/widowers | **1 2** | | k) Support for orphans | **1 2** | | l) Spiritual support | **1 2** | | m) Other 1 2 | | | Specify_________________________ | | | |  | | --- | |  | | [__] | | [__] | | [__] | | [__][__] | | [__] | | [__] | | [__] | | [__] | | [__] | | [__] | | [__] |   **[__]** |  |
| 114 | Does this facility have a working relationship with any HIV/AIDS service providers (government/ NGO) | Yes 1 No  **2** | [__] | If 2, 0r 3, go to 116 |
| 115 | What is the nature of this relationship?  **Record all that apply** | | Yes=1; No=2 |  | | --- | --- | |  | Yes No | | a) Referral | **1 2** | | b) Supervision | **1 2** | | c) Training | **1 2** | | d) Supply of commodities | **1 2** | | e) Laboratory services | **1 2** | | f) Other | **1 2** | | Specify_____________________ | | | Specify2 ______________________ | | | |  | | --- | |  | | [__] | | [__] | | [__] | | [__] | | [__] | | [__] | |  |
| 116 | Do you refer patients/clients who are HIV positive to any of the following facilities?  **(R**ead out each and circle which one applies) | | Yes=1; No=2 |  | | --- | --- | |  | Yes No | | National Referral Hospital (UTH) | **1 2** | | b) Provincial Hospital | **1 2** | | c) District Hospital | **1 2** | | d) Mission Hospital | **1 2** | | e) Health Centre/clinic | **1 2** | | f) Health Post | **1 2** | | g) NGO | **1 2** | | h) CBO | **1 2** | | i) Fixed Outreach | 1. **2** | | j)Home-based care | **1 2** | | k) Other | **1 2** | | Specify_____________________ | | | |  | | --- | |  | | [__] | | [__] | | [__] | | [__] | | [__] | | [__] | | [__] | | [__] | | [__] | | [__] |  [__] |  |
| 117 | For what specific services do you refer the patients/clients?  **(Read out and circle 1 or 2 )** | | Yes=1; No=2 |  | | --- | --- | |  | Yes No | | a) Home-based care | **1 2** | | b) VCT | **1 2** | | c) CD4 Count | **1 2** | | d) ART | **1 2** | | e) PMTCT | **1 2** | | f) Support Services | **1 2** | | g) Management of OIs | **1 2** | | h) Non-HIV-related services | **1 2** | | i) Other (HIV-related services) | **1 2** | | Specify1 **_____________________** | | | Specify2______________________ | | | |  | | --- | |  | | [__] | | [__] | | [__] | | [__] | | [__] | | [__] | | [__] | | [__] | | [__] | |  | |  |
| 118 | Does this facility distribute **condoms?** | Yes 1 No **2** | [___] |  |
| 119 | Does this facility have a pharmacy? | Yes 1 No **2** | [___] | If no, go to 121 |
| 120 | When did you first have the pharmacy at this facility? | Year __________ Month__________ |  |  |
| 121 | Does this facility provide laboratory services? | Yes 1 No **2** | [___] | If no, end here |
| 122 | When did you start providing laboratory services? | Year __________ Month__________ |  |  |
| I would now like to find out more information regarding the following:   - Human Resources - Laboratory services - Pharmacy services - Records/register review for outpatient/inpatient services, ART, VCT, PMTCT, condom numbers.   Please let me know if you are the most appropriate person to speak with or if I should speak with the person in charge of these departments.  **Interviewer:** move to sections mentioned above, asking the manager to introduce you to the most appropriate person. | | | | |
