# Supplementary material for: Task sharing in Zambia: HIV service scale-up compounds the human resource crisis
Source: BMC Health Serv Res. 2010 Sep 17;10:272. doi: 10.1186/1472-6963-10-272 (PMC2955013; doi:10.1186/1472-6963-10-272)
Supplement: Additional file 3 — Health Facility Survey - Human Resources. A structured questionnaire, administered to health facility managers, to ascertain health worker categories, trends over time and staff allocations to services. [file 1472-6963-10-272-S3.DOC]

**Global HIV/AIDS Initiatives in Zambia**

**Health Facility Survey - Human Resources**

**Phase 2 – June 2008**

### Questionnaire No.: [________] *Office use only* Date of Interview (dd/mm/yyyy): [____/____/________] Name of Facility ____________________________­________

Location of Facility ____________________________________

Result Code **1** Completed

**2** Respondent not available

**3** Refused **[__]**

**4** Partially completed

**5** Other

Specify____________________________

Research Interviewer (RI) **[____]**

**RI Sign.___________ Checked by Supervisor_____________ Date___/___/_______**

| **Section A: Human Resources** |
| --- |
| **Find the person in charge of human resources. If he/she is not present, ask to speak with the person who is most knowledgeable about Human Resource services in the facility. Ask the Manager to introduce you to this person.** |

| **101** | Are you the person in charge of human resources at this facility? | | | | Yes **1**  No **2** | **[__]** |  |
| --- | --- | --- | --- | --- | --- | --- | --- |
| I would like to find out how many health workers have been employed in this facility over the last three years.  **(Total number of staff employed *by profession )*** | | | | | |  |  |
|  | | **102. Total currently employed** | **103. Total employed**  **Jan 2008** | **104. Total employed Jan 2007** | **105. Total employed Jan 2006** | **106. Total employed Jan 2005** | **107. Total employed Jan 2004** |
| **Total number of staff involved in the provision of *health services*** | |  |  |  |  |  |  |
| a. Physician (general) | |  |  |  |  |  |  |
| b. Physician (specialist) | |  |  |  |  |  |  |
| c. Clinical officers | |  |  |  |  |  |  |
| d. Nurse (ZRN) | |  |  |  |  |  |  |
| e. Nurse (ZEN) | |  |  |  |  |  |  |
| f. Laboratory technician | |  |  |  |  |  |  |
| g. Laboratory Assistant | |  |  |  |  |  |  |
| h. Pharmacists | |  |  |  |  |  |  |
| i. Pharmacy Assistant | |  |  |  |  |  |  |
| j. Dedicated HIV counselors | |  |  |  |  |  |  |
| k. Records/registry clerk | |  |  |  |  |  |  |
| l. Others | |  |  |  |  |  |  |
| **Total number of *ALL* Staff** | |  |  |  |  |  |  |

| ***108. How many health workers are involved in providing VCT services at this facility?*** | | | | | | | |
| --- | --- | --- | --- | --- | --- | --- | --- |
|  |  | **Current** | **Jan 2008** | **Jan 2007** | **Jan 2006** | ***Jan 2005*** | ***Jan 2004*** |
| a. | Full-time provision of counseling services for HIV |  |  |  |  |  |  |
| b. | Part-time provision of counselling services for HIV services. |  |  |  |  |  |  |
| c. | Laboratory tests for HIV Alone |  |  |  |  |  |  |
| d. | Laboratory tests for HIV and non-  HIV services |  |  |  |  |  |  |
| e. | ***Total*** |  |  |  |  |  |  |
| ***109. How many health workers are involved in providing ART services at this facility?*** | | | | | | | |
|  |  | **Current** | **Jan 2008** | **Jan 2007** | ***Jan 2006*** | ***Jan 2005*** | ***Jan 2004*** |
| a. | ART alone |  |  |  |  |  |  |
| b. | ART and non-HIV services |  |  |  |  |  |  |
| c. | Total |  |  |  |  |  |  |
| ***110. How many health workers are involved in providing PMTCT services at this facility?*** | | | | | | | |
|  |  | **Current** | **Jan 2008** | **Jan 2007** | ***Jan 2006*** | ***Jan 2005*** | ***Jan 2004*** |
| a | PMTCT alone |  |  |  |  |  |  |
| B | PMTCT and non-HIV services |  |  |  |  |  |  |
| C | ***Total*** |  |  |  |  |  |  |

**Section B. Staff Training and Incentives**

| 111 | Have staff at this facility received any kind of training whilst employed at this facility in 2007? | | Yes **1**  No **2** | | | | **[__]** | ***If no, skip to 116*** |
| --- | --- | --- | --- | --- | --- | --- | --- | --- |
| 112 | How many staff have received training in 2007? | | Record number: _____________ | | | |  |  |
| 113 | What type of training were received in 2007  ***(Read out and record number)*** | | **1 = Yes, 2 = No No. trained**   | 1. HIV testing | **1 2** | **[__]** | **[_____]** | | --- | --- | --- | --- | | 1. HIV counselling | **1 2** | **[__]** | **[_____]** | | 1. ART | **1 2** | **[__]** | **[_____]** | | 1. PMTCT | **1 2** | **[__]** | **[_____]** | | 1. Child Health | **1 2** | **[__]** | **[_____]** | | 1. Maternal Health | **1 2** | **[__]** | **[_____]** | | 1. Family Planning | **1 2** | **[__]** | **[_____]** | | 1. TB | **1 2** | **[__]** | **[_____]** | | 1. Malaria | **1 2** | **[__]** | **[_____]** | | 1. Other | **1 2** | **[__]** | **[_____]** |   Specify1____________________ **[_____]**  Specify2____________________ **[_____]**  Specify3____________________ **[_____]** | | | | **[_____]**  **[_____]**  **[_____]**  **[_____]**  **[_____]**  **[_____]**  **[_____]**  **[_____]**  **[_____]**  **[_____]**  **[_____]**  **[_____]**  **[_____]** |  |
| 114 | Do incentives/job benefits exist for staff providing the following services at this facility? |  | | **Yes No** | **Financial**  **Yes No** | **Non-financial**  **Yes No** | **[__]**  **[__]**  **[__]**  **[__]**  **[__]**  **[__]**  **[__]**  **[__]** |  |
|  |  | a) ART  b) VCT  c) PMTCT  d) TB  e) Malaria  f) Family Planning  g) Child Health  h) Maternal Health | | **1 2 [__]**  **1 2 [__]**  **1 2 [__]**  **1 2 [__]**  **1 2 [__]**  **1 2 [__]**  **1 2 [__]**  **1 2 [__]** | **1 2 [__]**  **1 2 [__]**  **1 2 [__]**  **1 2 [__]**  **1 2 [__]**  **1 2 [__]**  **1 2 [__]**  **1 2 [__]** | **1 2**  **1 2**  **1 2**  **1 2**  **1 2**  **1 2**  **1 2**  **1 2** |  |  |
| 115 | Do staff receive greater financial incentives for delivering HIV/AIDS related services than for non-HIV/AIDS related services? | | | Yes 01  No 02  Don’t know 03 | | | **[__]** |  |
| 116 | If some facility staff are on training, does this increase workload on staff in the facility? | | | Yes **1**  No **2** | | | **[__]** | **If No, end here** |
| 117 | Are any incentives/compensation provided to staff who remain in post. | | | Yes **1**  No **2** | | | **[__]** | **If No, end here** |
| 118 | What is the nature of these incentives | | | Financial 1 Non-financial **2** | | | **[__]** |  |

**Research Interviewer: Thank the Respondent before leaving**
